# IL-33 regulates Müller cell-mediated retinal inflammation and neurodegeneration in diabetic retinopathy

**DOI:** 10.1242/dmm.050174

**Published:** 2023-09-06

**Authors:** Josy Augustine, Sofia Pavlou, Kevin Harkin, Alan W. Stitt, Heping Xu, Mei Chen

**Affiliations:** Wellcome-Wolfson Institute for Experimental Medicine, School of Medicine, Dentistry and Biomedical Sciences, Queen's University Belfast, Belfast BT9 7BL, Northern Ireland, UK

**Keywords:** Cytokines, Diabetes, Interleukin-33, Neurotrophins, Neurovascular unit, Retina

## Abstract

Diabetic retinopathy (DR) is characterised by dysfunction of the retinal neurovascular unit, leading to visual impairment and blindness. Müller cells are key components of the retinal neurovascular unit and diabetes has a detrimental impact on these glial cells, triggering progressive neurovascular pathology of DR. Amongst many factors expressed by Müller cells, interleukin-33 (IL-33) has an established immunomodulatory role, and we investigated the role of endogenous IL-33 in DR. The expression of IL-33 in Müller cells increased during diabetes. Wild-type and *Il33^−/−^* mice developed equivalent levels of hyperglycaemia and weight loss following streptozotocin-induced diabetes. Electroretinogram a- and b-wave amplitudes, neuroretina thickness, and the numbers of cone photoreceptors and ganglion cells were significantly reduced in *Il33^−/−^* diabetic mice compared with those in wild-type counterparts. The *Il33^−/−^* diabetic retina also exhibited microglial activation, sustained gliosis, and upregulation of pro-inflammatory cytokines and neurotrophins. Primary Müller cells from *Il33^−/−^* mice expressed significantly lower levels of neurotransmitter-related genes (*Glul* and *Slc1a3*) and neurotrophin genes (*Cntf*, *Lif*, *Igf1* and *Ngf*) under high-glucose conditions. Our results suggest that deletion of IL-33 promotes inflammation and neurodegeneration in DR, and that this cytokine is critical for regulation of glutamate metabolism, neurotransmitter recycling and neurotrophin secretion by Müller cells.

## Teaser

**Summary:** IL-33 is upregulated in Müller cells of murine retina during experimental diabetes, and deletion of IL-33 promotes gliosis, inflammation and neurodegeneration during streptozotocin-induced diabetic retinopathy.

## INTRODUCTION

Diabetic retinopathy (DR) is a progressive sight-threatening disease that alters normal retinal cell interactions and leads to glial cell dysfunction, impaired neuronal function and vascular abnormalities (Duh et al., 2017; Stitt et al., 2016; Antonetti et al., 2021). Subsequent visual impairment due to DR is increasing globally and represents a significant cost for healthcare systems and the economy (Flaxman et al., 2017; Ong et al., 2023). Recent comprehensive systematic reviews and meta-analyses demonstrate that one-third of patients with diabetes worldwide are affected by DR, which increases noticeably after the age of 60 years owing to a longer duration of diabetes, with no intergender difference for its prevalence (Teo et al., 2021; Hashemi et al., 2022; Lundeen et al., 2023).

The diabetic milieu detrimentally impairs several components of the retinal neurovascular unit (NVU), including the vascular endothelium, neurons, and glial and immune cells, in the pathogenesis of DR (Simó et al., 2018; Antonetti et al., 2021). NVU dysregulation is an early event observed in patients and animal models of DR, which results in impaired neurovascular coupling, loss of autoregulation and control of blood flow, as well as disruption of the inner blood-retinal barrier (BRB) (Augustine et al., 2021; Duh et al., 2017; O'Hare et al., 2022; Antonetti et al., 2021). Diabetes inflicts early damage upon the retina through mechanisms not addressed by the currently available treatments targeting the late-stage pathology of DR, such as neovascularisation. Thus, there is an unmet need for therapeutic targets to focus on earlier stages of NVU disruption in DR (Sohn et al., 2016; Stitt et al., 2016), in which loss of normal homeostasis leads to neuroinflammation (Tang and Kern, 2011; Altmann and Schmidt, 2018), glial cell dysfunction (Reichenbach and Bringmann, 2010; Coughlin et al., 2017; Duh et al., 2017) and degenerative pathology (Simó et al., 2018) across multiple retinal cells, as demonstrated in clinical and experimental animal models.

Müller cells span the entire thickness of the neural retina and have a key role in homeostasis of the neuropile through regulation of substrates and waste products of metabolism, maintenance of the BRB and fluid regulation (Reichenbach and Bringmann, 2013; Bringmann et al., 2009). Müller cells achieve this via production of antioxidants (glutathione) and secretion of neurotrophins [brain-derived neurotrophic factor (BDNF), ciliary neurotrophic factor (CNTF), glial cell-derived neurotrophic factor (GDNF), insulin-like growth factor 1 (IGF1), leukaemia inhibitory factor (LIF), nerve growth factor (NGF) and neurotrophin 3 (NTF3)] in close association with other component cells of the NVU (Fu et al., 2015; Reichenbach and Bringmann, 2013; Boss et al., 2017; Chen et al., 2022). These glial cells mediate neurovascular coupling, homeostasis of the extracellular space volume under intense neuronal activity, modulation of neuronal activity by release of neuroactive signalling molecules, and regulation of the synaptic activity in the inner retina by uptake of glutamate through glutamate aspartate transporter (GLAST, encoded by *Slc1a3*) (Reichenbach and Bringmann, 2013; Fu et al., 2015; Bringmann et al., 2013).

Diabetes leads to dysfunction of Müller cells and they assume a reactive phenotype, characterised by upregulation of glial fibrillary acidic protein (GFAP), inflammatory cytokines [IL-1β, IL-6, IL-8 and TNFα (encoded by *Il1b*, *Il6*, *Cxcl15* and *Tnf*, respectively)] and neurotrophins (BDNF, CNTF, GDNF, NGF and NTF3), and downregulation of glutamine synthetase (GS, encoded by *Glul*), to mitigate tissue damage prior to clinical manifestations of DR (Reichenbach and Bringmann, 2010; Takeuchi et al., 2015; Boss et al., 2017; McDowell et al., 2018). Transcriptional changes in activated Müller cells contribute to pathological changes in DR, including glutamate metabolic dysfunction, neuronal apoptosis, BRB breakdown and microvascular lesions (Simó et al., 2018; Duh et al., 2017; Coughlin et al., 2017; Reichenbach and Bringmann, 2010). Furthermore, evidence suggests that there is impaired communication between Müller cells and microglia via the CD40-ATP-P_2_X_7_ pathway, which provokes phenotypic switching of microglia to an activated state, thus accelerating neuroinflammation (Portillo et al., 2017). Recent studies have demonstrated that interleukin-33 (IL-33), an immunomodulatory cytokine, is predominantly expressed in human macula and rodent retina, where it regulates disease-linked inflammatory responses (Xi et al., 2016; Augustine et al., 2019; Theodoropoulou et al., 2017). However, the role of IL-33 in the pathogenesis of DR remains unelucidated.

IL-33 is an innate immunomodulatory cytokine that belongs to the IL-1 cytokine family and resides within the nuclei of various cell types (Clare et al., 2021; Liew et al., 2016; Schmitz et al., 2005). Under cellular and tissue homeostasis, endogenous IL-33 negatively affects gene transcription as a nuclear protein in an intracrine fashion by sequestration of NF-κB, leading to the repression of gene expression to dampen pro-inflammatory pathways (Martin and Martin, 2016; Ali et al., 2011). In contrast to other IL-1 cytokine family members, IL-33 lacks a classical signal peptide for secretion from cells and is bioactive at full length. IL-33 is released upon cell damage or stress as an alarmin to signal via the ST2 (IL1RL1) receptor, which is widely expressed on immune cells to promote host resistance and type 2 allergic immunity (Liew et al., 2016). It induces signalling cascades through the Toll/interleukin-1 receptor (TIR), thereby activating downstream pathways inducing Th2-type immunity, which can be pro-inflammatory or anti-inflammatory (Schmitz et al., 2005; Martin and Martin, 2016; Liew et al., 2016).

Previously, we have shown that the protective role of endogenous IL-33 expressed in Müller cells alleviates retinal inflammation, reflected through reduced gliosis, macrophage activation and neurodegeneration in a mouse model of retinal detachment (Augustine et al., 2019). Similar protective roles have been reported in autoimmune uveoretinitis (Barbour et al., 2014). Furthermore, it has been shown that endogenous IL-33 exerts control over mitochondrial respiration in the retinal pigment epithelium (RPE) by facilitating oxidative pyruvate catabolism to maintain homeostasis, which thus confirms the role of IL-33 as a key metabolic checkpoint regulator in the RPE, exerting profound effects on retinal metabolism (Scott et al., 2021). In addition, the protective capability of exogenous IL-33 against RPE loss and for retinal metabolic homeostasis has been shown in a dysregulated immune-mediated insidious model of outer retinal degeneration (Clare et al., 2020). IL-33 also limits pathology by suppressing the activation and migration of choroidal endothelial cells and fibroblasts in the context of choroidal neovascularisation (Theodoropoulou et al., 2017). Conversely, it has been shown that the release of IL-33 from Müller cells contributes to the pathogenesis of age-related macular degeneration (AMD) by triggering an inflammatory response and photoreceptor degeneration (Xi et al., 2016).

In the current study, we investigated the role of endogenous IL-33 in the pathogenesis of DR. Using streptozotocin (STZ)-induced diabetes in wild-type C57BL/6J (WT) and *Il33* knockout (*Il33^−/−^*) mice, retinal inflammation, micro- and macro-glial activation, electrophysiological function, retinal thinning, neuronal loss, and development of acellular capillaries were assessed, with parallel analysis of cell-specific molecular pathways *in vivo* and *in vitro*. The deletion of endogenous IL-33 accelerated neuroinflammatory, gliotic and degenerative pathology during diabetes. Our results suggest that IL-33 is critical for the immunomodulatory function of Müller cells through regulation of glutamate metabolism, neurotransmitter recycling and secretion of neurotrophins. Thus, our data reveal that Müller cells regulate expression of IL-33 to dampen the neuroinflammatory, glial activation and degenerative pathology of DR.

## RESULTS

### IL-33 expression is upregulated in Müller cells during diabetes

The specificity of *IL33* expression was examined in the human retinal single-cell RNA sequencing (scRNA-seq) dataset GSE196235 deposited in the Gene Expression Omnibus (GEO; National Center for Biotechnology Information) database, from eight post-mortem retinas of four individuals who had no history of eye disease (Wang et al., 2022). Single-cell transcriptomic analysis of the human retina was used to identify major retinal cell types, and 22 clusters were resolved and assigned to 13 cell types according to their corresponding gene-expression signatures (Fig. S1A,B). *IL33* expression was dominant in the Müller cell clusters in comparison with other retinal cell types (Fig. S1C,D).

**Fig. 1.**
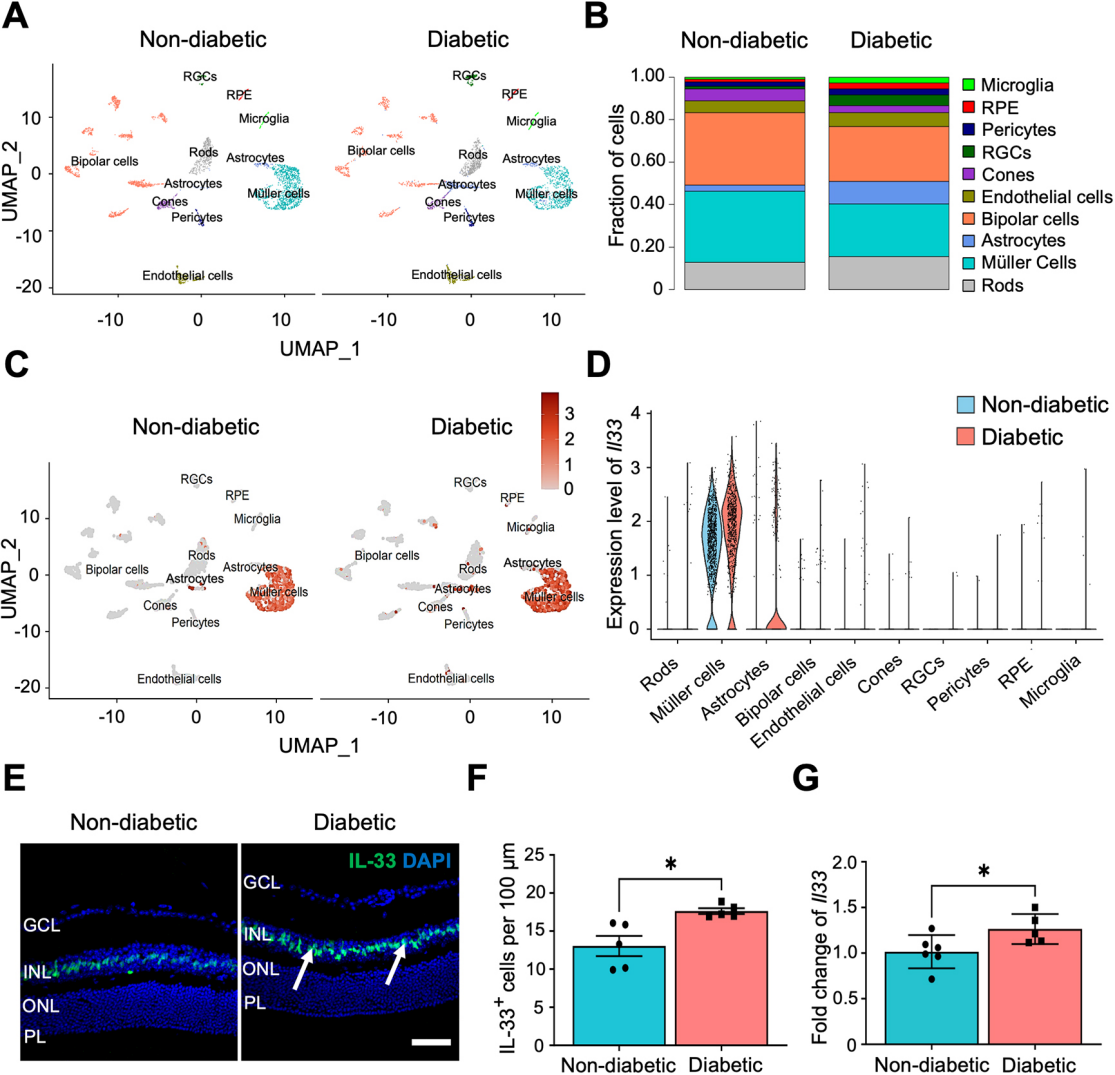
**Expression of IL-33 in non-diabetic and diabetic murine retina.** (A) ScRNA-seq data showing the uniform manifold approximation and projection (UMAP) plot of ten cell clusters in non-diabetic and diabetic retinas. (B) Fraction of different cell types in the non-diabetic and diabetic murine retinas as determined by scRNA-seq, calculated from absolute counts out of 21,824. (C) The global expression changes of *Il33* are exhibited in the UMAP plots. (D) Violin plots display the dominant expression of *Il33* in the Müller cell cluster of retinal cells. (E) Representative images showing the expression of IL-33 (green, arrows) and DAPI (blue) in retinal cryosections of non-diabetic and diabetic mice. Scale bar: 50 μm. (F) Bar graph showing the average number of IL-33^+^ cells normalised to 100 μm of retina length. *n*=5 mice per experimental group. (G) Bar graph showing *Il33* mRNA expression in non-diabetic and diabetic retinas relative to *Actb* expression, assessed by RT-qPCR. These data are also plotted in Fig. S3B for the WT non-diabetic and diabetic retinas, as a ratio of *Il33* to *Actb* expression. *n*=5-6 retinas per experimental condition. Data show the mean±s.e.m. Unpaired two-tailed Student's *t*-test (F,G) was used; *P<0.05. GCL, ganglion cell layer; INL, inner nuclear layer; ONL, outer nuclear layer; PL, photoreceptor layer; RGCs, retinal ganglion cells; RPE, retinal pigment epithelium.

The retinal expression of *Il33* was then investigated in non-diabetic and diabetic mice (6 months of diabetes) by scRNA-seq, immunohistochemistry and real-time quantitative PCR (RT-qPCR). Single-cell transcriptomic analysis was performed on the dataset GSE178121 from the GEO database (Sun et al., 2021). The retinal cells were clustered into rods, Müller cells, cones, microglia, bipolar cells, amacrine cells, astrocytes, endothelial cells, retinal ganglion cells and RPE cells (Fig. 1A). The relative abundance of cell types was calculated, and an increase in the population of microglia, astrocytes and endothelial cells, and a decrease in the population of Müller cells and bipolar cells were observed in the diabetic retina in comparison with their abundance in the non-diabetic retina (Fig. 1B). The expression of *Il33* increased globally in the diabetic retina compared with that in the non-diabetic retina (Fig. 1C), with predominant expression in Müller cells in both non-diabetic (88.4%) and diabetic retinas (91.6%) (Fig. 1D). In addition, the number of IL-33^+^ cells in the inner nuclear layer (INL) of the diabetic retina was significantly higher when compared with that in the non-diabetic retina (Fig. 1E,F). We have previously shown that IL-33 is predominately expressed in Müller cells of the INL, when co-stained with the Müller-specific marker GS (Augustine et al., 2019). Subsequently, the increased expression of *Il33* in the diabetic retina was confirmed by RT-qPCR after 6 months of diabetes (Fig. 1G).

We then investigated the effect of diabetes on the gene expression of major cytokines, chemokines and neurotrophins in Müller cell and microglia. The expression of *Il33*, *Il18*, *Vim* and *Hif1a* was significantly upregulated in Müller cells of the diabetic retina when compared with that of the non-diabetic retina (Fig. S2A), and this was accompanied by the upregulation of *Gfap*, *Bdnf*, *Lif*, *Igf1* and *Nos3* (Fig. S2B). Additionally, the expression of *Hmox1*, *Ccl2*, *Tgfb* (*Tgfb1*) and *Tnf* was significantly increased in microglia of the diabetic retina (Fig. S2C). Autophagy malfunction is known to be critically involved in the pathogenesis of DR, including the damage of the NVU (Yang et al., 2023). Surprisingly, we did not detect any significant changes in autophagy-related genes in Müller cells.

### IL-33 deficiency results in upregulation of inflammatory cytokines, sustained retinal inflammation and Müller gliosis during diabetes

**Fig. 2.**
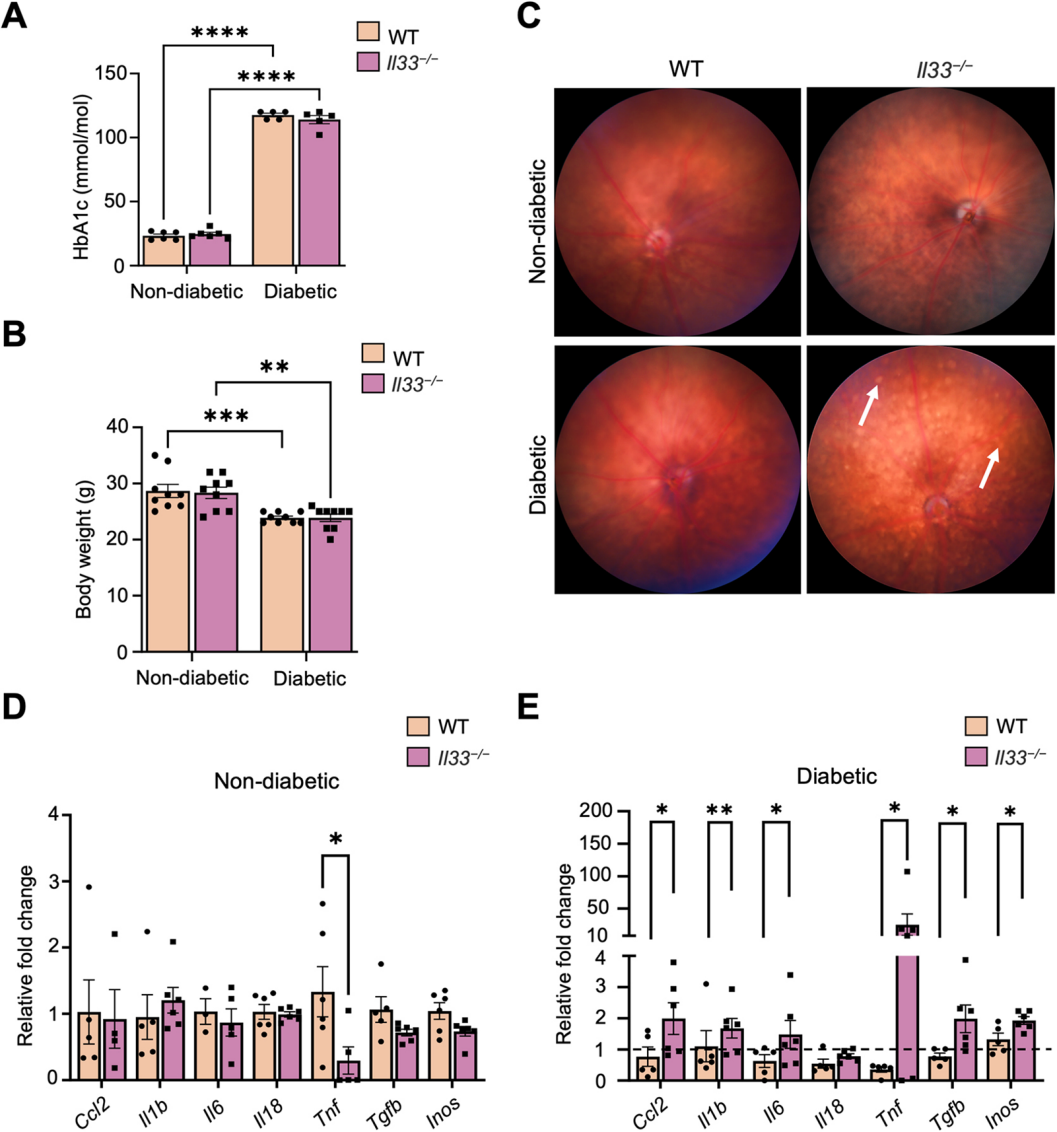
**Characterisation of diabetes in WT and *Il33^−/−^* mice and increased pro-inflammatory cytokine expression in *Il33^−/−^* diabetic retinas after 6 months.** (A) HbA1c levels recorded at the time of euthanasia from experimental groups of WT and *Il33^−/−^* non-diabetic and diabetic mice. *n*=6-10 animals per group. (B) Body weights recorded at the time of euthanasia from experimental groups of WT and *Il33^−/−^* non-diabetic and diabetic mice. *n*=6-10 animals per group. (C) Representative fundus images showing the interior surface of WT and *Il33^−/−^* eyes from non-diabetic and diabetic mice. Yellow exudate-like depositions (arrows) around the retinal vasculature and optic disc in the *Il33^−/−^* diabetic eye can be seen. *n=*5-6 mice per experimental condition. (D) Expression levels of pro-inflammatory cytokines and chemokines in non-diabetic WT and *Il33^−/−^* retinas. mRNA expression was assessed by RT-qPCR relative to *Rn18s* expression. (E) Expression levels of pro-inflammatory cytokines and chemokines in diabetic WT and *Il33^−/−^* retinas, compared with non-diabetic retinas from the same strain. Fold change values above the dotted line suggest increased response with diabetes and fold change values below the dotted line suggest decreased response with diabetes. mRNA expression was assessed by RT-qPCR relative to *Rn18s* expression. *n=*5-6 retinas per experimental condition. Data show the mean±s.e.m. Two-way ANOVA (A,B) and unpaired two-tailed Student's *t*-test (D,E) were used; *P<0.05; **P<0.01; ***P<0.001; ****P< 0.0001.

To understand the role of IL-33 in DR, we induced diabetes in WT and *Il33^−/−^* mice by STZ injection. The deletion of IL-33 was confirmed by immunohistochemistry and RT-qPCR in non-diabetic and diabetic retinas of *Il33^−/−^* mice (Fig. S3A,B). After 6 months of diabetes induction, a significant elevation of glycated haemoglobin (HbA1c) (>90 mmol/mol for both strains) (Fig. 2A) and loss of body weight (<25 g for both strains) (Fig. 2B) were observed in diabetic mice compared with non-diabetic counterparts. However, no significant difference in HbA1c levels or body weight were observed between WT and *Il33^−/−^* mice under non-diabetic or diabetic conditions (Fig. 2A,B).

Evaluation of the fundus revealed multiple yellow exudate-like depositions in the retinas of *Il33^−/−^* diabetic mice, but not in the retinas of WT and non-diabetic counterparts (Fig. 2C). Previous studies by our group and others have shown that these yellowish deposits are activated subretinal IBA-1 (encoded by *Aif1*)^+^ CD68^+^ P2RY12^+^ microglial cells (Luhmann et al., 2009; Chen et al., 2011; Aredo et al., 2015). RT-qPCR analyses showed that *Il33^−/−^* non-diabetic retinas had significantly lower levels of *Tnf* compared with those in WT counterparts (Fig. 2D); the expression of other inflammatory genes was comparable between *Il33^−/−^* and WT retinas. However, 6 months after the induction of diabetes, the expression levels of *Ccl2*, *Il1b*, *Il6*, *Tnf*, *Tgfb* and *Inos* (*Nos2*) were significantly higher in the *Il33^−/−^* retina than in the WT retina (Fig. 2E). The retinal expression level of *Il18* was not affected by diabetes in both WT and *Il33^−/−^* mice (Fig. 2E).

**Fig. 3.**
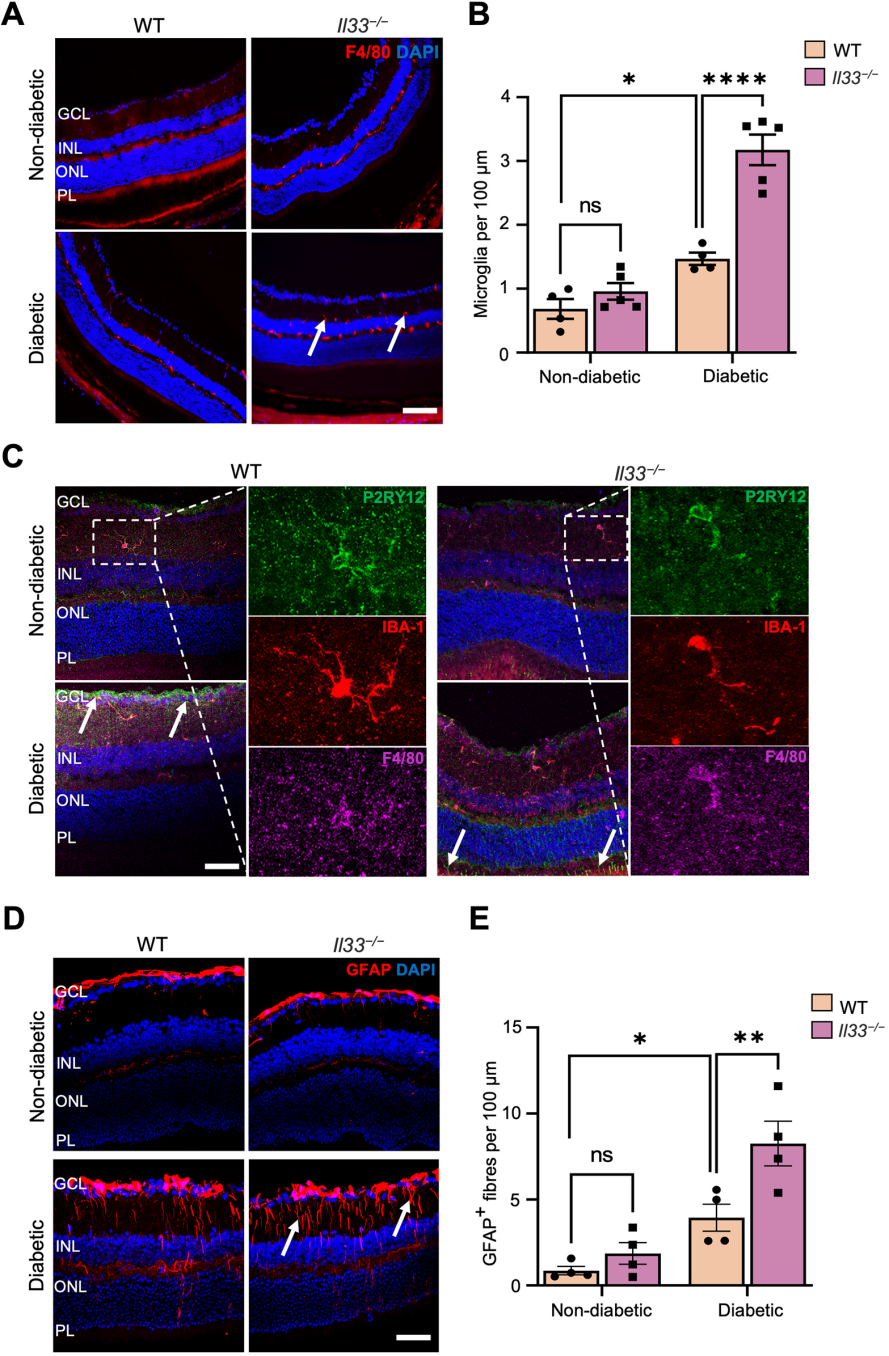
**Deletion of IL-33 results in microglial activation and gliosis in the retina after 6 months of diabetes.** (A) Representative images of retinal cryosections from experimental groups of WT and *Il33^−/−^* non-diabetic and diabetic mice immunostained for F4/80 (red, arrows), and counterstained with DAPI (blue). (B) Bar graphs showing the average number of microglia in the IPL and OPL normalised to 100 μm of retina length. *n*=4-5 mice per experimental group. (C) Representative images of retinal cryosections from WT and *Il33^−/−^* non-diabetic and diabetic mice, showing co-immunostaining for P2RY12 (green, arrows and inset), IBA-1 (red, inset) and F4/80 (magenta, inset), and counterstained with DAPI (blue). (D) Representative images of retinal cryosections from WT and *Il33^−/−^* non-diabetic and diabetic mice immunostained for GFAP (red, arrows) and counterstained with DAPI (blue). (E) Bar graphs showing the average number of GFAP^+^ fibres and normalised to 100 μm of retina length. *n*=4-5 mice per experimental group. Data show the mean±s.e.m. Two-way ANOVA (B,E) was used; ns, not significant; *P<0.05; **P<0.01; ****P<0.0001. GCL, ganglion cell layer; INL, inner nuclear layer; ONL, outer nuclear layer; PL, photoreceptor layer. Scale bars: 100 μm (A); 50 μm (C,D).

F4/80 (encoded by *Adgre1*)^+^ cells were detected in the ganglion cell layer (GCL), the inner plexiform layer (IPL) and the outer plexiform layer (OPL) of the WT and *Il33^−/−^* non-diabetic control and diabetic retinas (Ozaki et al., 2022). A significantly higher number of F4/80^+^ cells was detected in the IPL and OPL of *Il33^−/−^* diabetic retinas compared with that in the WT counterpart (Fig. 3A,B). Further phenotypic study showed that these cells co-express IBA-1 and P2RY12 (Fig. 3C), suggesting that they are active microglial cells. Interestingly, some photoreceptors and retinal nerve fibre layers (NFLs) were also positive for P2RY12, particularly in diabetic eyes (arrows in Fig. 3C). GFAP was detected in the NFL, GCL, IPL and INL of WT and *Il33^−/−^* non-diabetic retinas (Fig. 3D), and the expression extended to the OPL and outer nuclear layer (ONL) in diabetic retinas (Fig. 3D). The number of GFAP^+^ fibres did not differ between non-diabetic WT and *Il33^−/−^* retinas (Fig. 3E), but was significantly higher in the *Il33^−/−^* diabetic retina compared with that in the WT counterpart (Fig. 3D,E). Taken together, our results suggest that deficiency of IL-33 leads to sustained inflammation, enhanced inflammatory cytokine expression and gliosis in the retina during diabetes.

### IL-33 deletion worsens diabetes-mediated reduction of retinal neurophysiological function

Previous studies have shown that diabetes reduces both a- and b-wave amplitudes of electroretinograms (ERGs) along with the kinetics of the oscillatory potentials (OPs) (Sergeys et al., 2019; Pavlou et al., 2019). In this study, there was no alteration in the amplitudes and implicit times of a-wave, b-wave and OPs among age-matched non-diabetic (6-month-old) and diabetic (for 3 months) WT and *Il33^−/−^* mice (Fig. S4A-G).

**Fig. 4.**
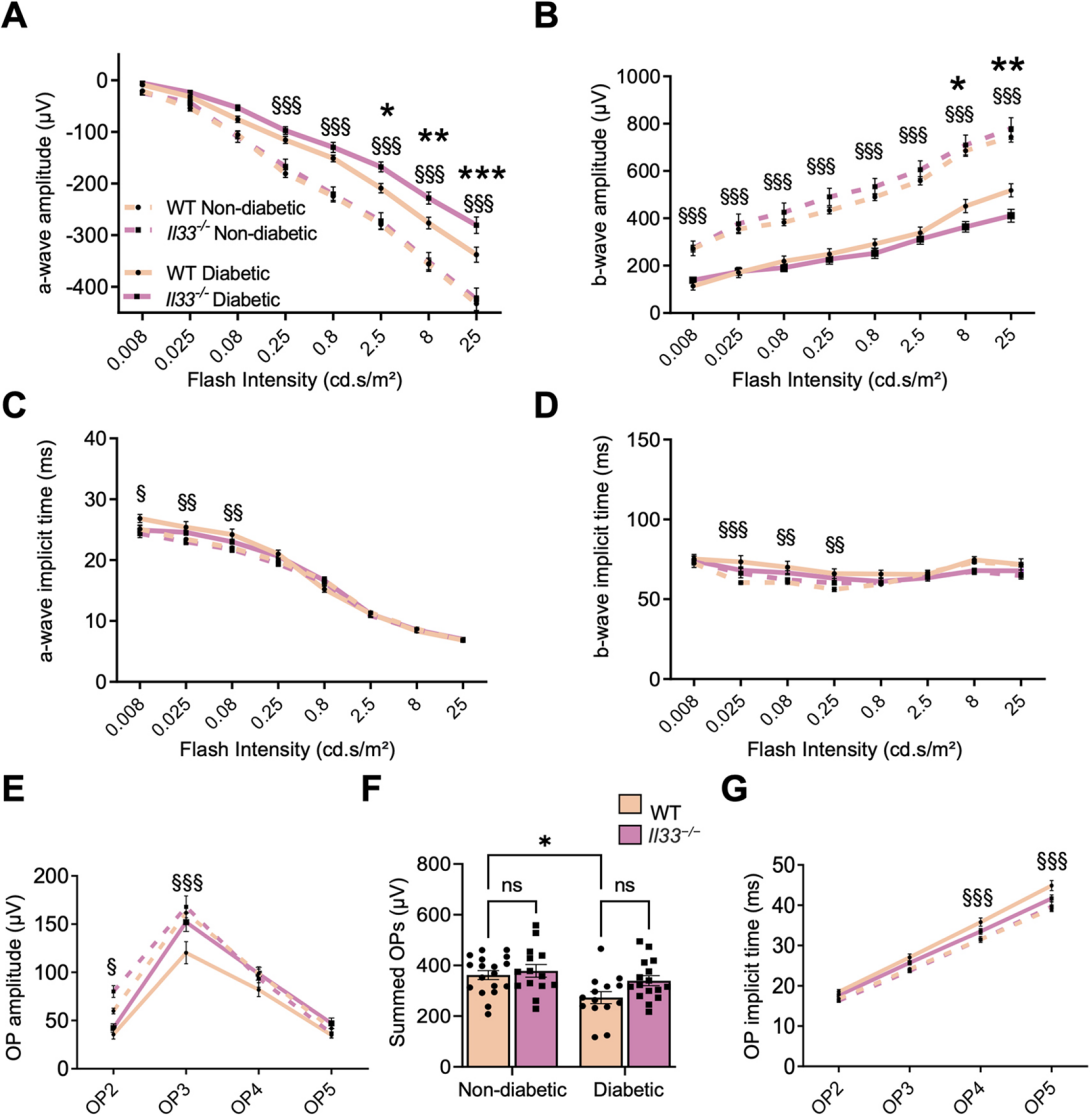
**IL-33 deficiency results in deterioration of retinal function and reduced ERG a- and b-wave response in the retina after 6 months of diabetes.** (A-D) Line graphs of average scotopic ERG a-wave (A), average scotopic ERG b-wave (B), average scotopic ERG a-wave implicit time (C) and average scotopic ERG b-wave implicit time (D) in the eyes of WT and *Il33^−/−^* non-diabetic and diabetic mice, quantified from 0.008 cd.s/m^2^ to 25 cd.s/m^2^. (E,G) Line graphs of average oscillatory potential (OP) amplitudes (E) and average OP implicit times (G) in the eyes of WT and *Il33^−/−^* non-diabetic and diabetic mice, quantified at 25 cd.s/m^2^. (F) Bar graph of summed OP amplitudes in the eyes of WT and *Il33^−/−^* non-diabetic and diabetic mice, quantified at 25 cd.s/m^2^. *n*=7-9 mice per experimental group. Data show the mean±s.e.m. Two-way ANOVA was used; ns, not significant; ^§^*P*<0.05, ^§§^*P*<0.01, ^§§§^*P*<0.001 between WT non-diabetic and diabetic eyes; *P<0.05, **P<0.01, ***P<0.001 between WT diabetic and *Il33^−/−^* diabetic eyes.

Furthermore, there were no differences in the amplitudes and implicit times of a-waves, b-waves and OPs between 9-month-old non-diabetic WT and *Il33^−/−^* mice (Fig. 4A-G). However, the amplitudes and implicit times of a-waves, b-waves and OPs were significantly decreased after 6 months of diabetes (9 months of age) in WT mice compared with those in the non-diabetic counterparts across a wide range of light stimulus intensities (Fig. 4A,B,F,G). The amplitudes of both a- and b-waves in 9-month-old *Il33^−/−^* diabetic mice (6 months of diabetes) were further reduced compared with those in WT counterparts (Fig. 4A,B). However, OPs and implicit times of a-waves and b-waves were similar between WT and *Il33^−/−^* diabetic mice (Fig. 4C-G). Taken together, our results suggest that deletion of IL-33 worsens diabetes-induced reduction of retinal electrophysiological functions.

### IL-33 deficiency worsens diabetes-induced retinal neurodegeneration

**Fig. 5.**
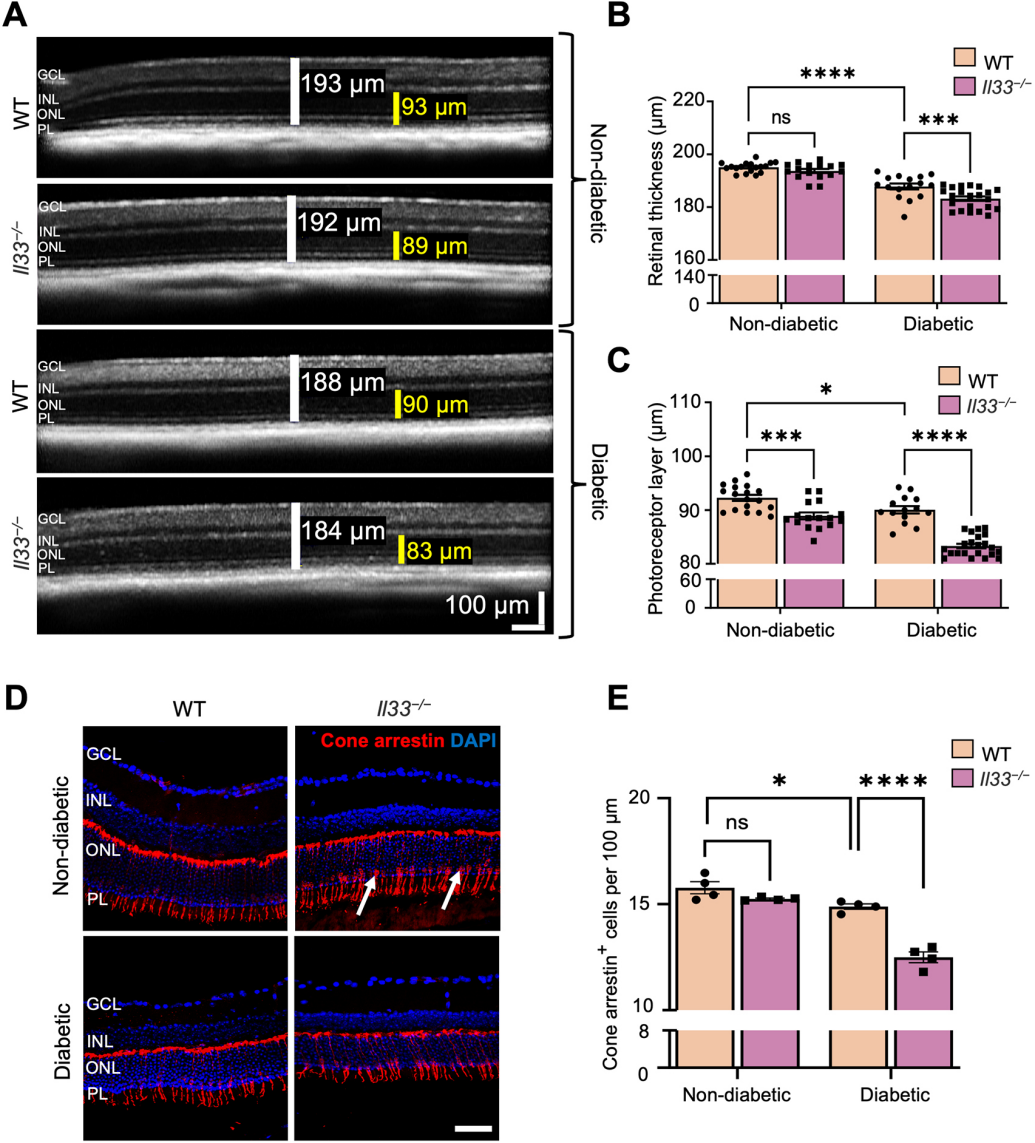
**Deletion of IL-33 results in reduced retinal and photoreceptor layer thickness and loss of cone cells in the retina after 6 months of diabetes.** (A) Representative images of SD-OCT measurements of total neuroretina (white bar) and photoreceptor layer (yellow bar) in the retinas of WT and *Il33^−/−^* non-diabetic and diabetic mice. Scale bar: 100 μm. (B,C) Bar graph showing the average total retinal thickness (B) and average photoreceptor layer thickness (C) in the retinas of WT and *Il33^−/−^* non-diabetic and diabetic mice. *n*=8-11 mice per experimental group. (D) Representative images of retinal cryosections from WT and *Il33^−/−^* non-diabetic and diabetic mice immunostained for cone arrestin (red, arrows) and counterstained with DAPI (blue). Scale bar: 50 μm. (E) Bar graph showing the average number of cone photoreceptors normalised to 100 μm of retina length. *n*=4-5 mice per experimental group. Data show the mean±s.e.m. Two-way ANOVA was used; ns, not significant; *P<0.05; ***P<0.001; ****P<0.0001. GCL, ganglion cell layer; INL, inner nuclear layer; ONL, outer nuclear layer; PL, photoreceptor layer.

**Fig. 6.**
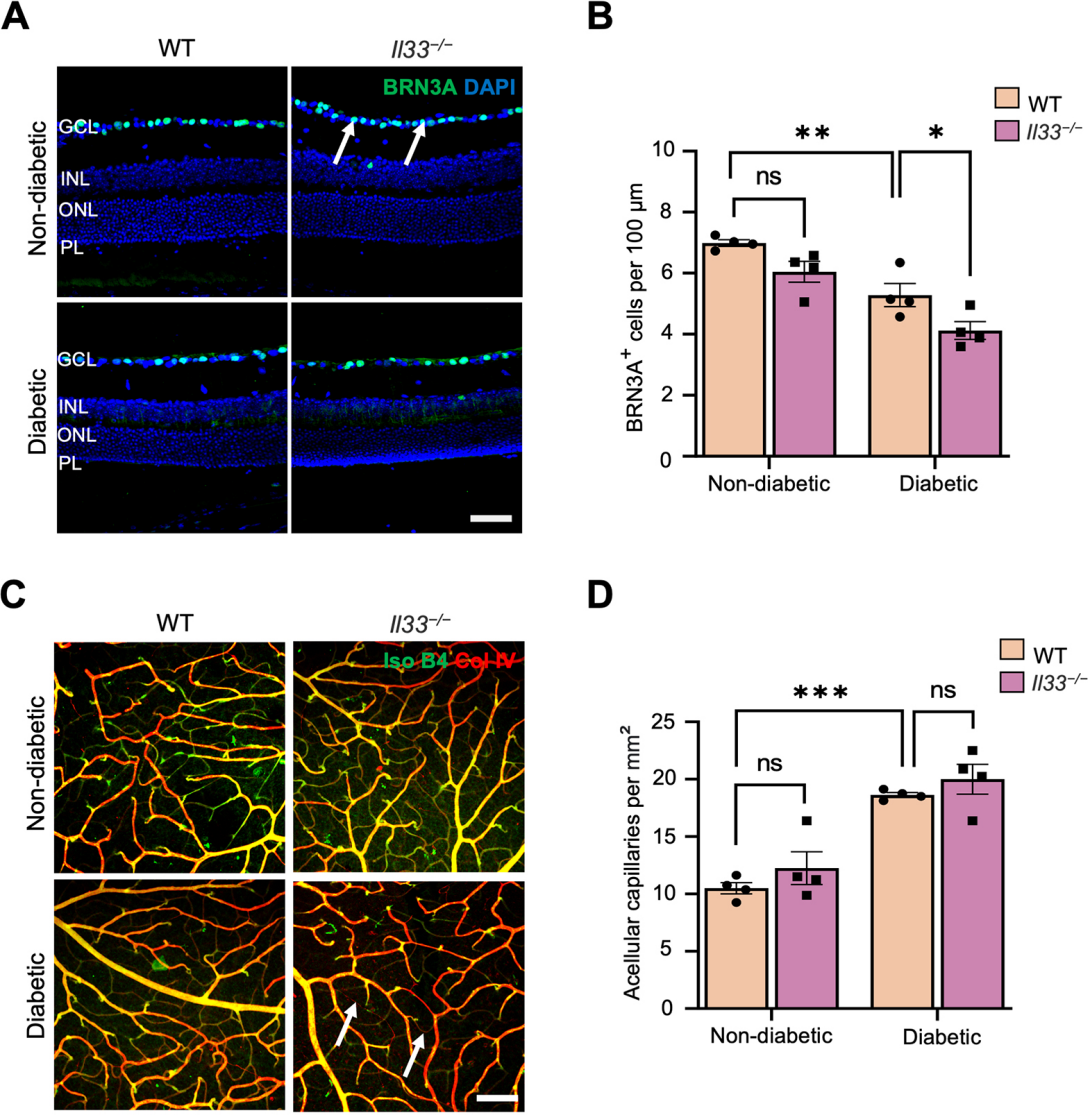
**Deletion of IL-33 results in loss of ganglion cells and no alteration of acellular capillaries in the retina after 6 months of diabetes.** (A) Representative images of retinal cryosections from WT and *Il33^−/−^* non-diabetic and diabetic mice immunostained for BRN3A^+^ ganglion cells (green, arrows) and counterstained with DAPI (blue). Scale bar: 50 μm. GCL, ganglion cell layer; INL, inner nuclear layer; ONL, outer nuclear layer; PL, photoreceptor layer. (B) Bar graph showing the average number of ganglion cells normalised to 100 μm of retina length. *n*=4-5 mice per experimental group. (C) Representative images of retinal flatmount staining from WT and *Il33^−/−^* non-diabetic and diabetic mice immunostained for isolectin B4 (green) and collagen IV (red, arrows). Scale bar: 100 μm. (D) Bar graph showing the average number of acellular capillaries normalised to 0.1 mm^2^ of retina. *n*=4 mice per experimental group and eight images per retinal flatmount. Data show the mean±s.e.m. Two-way ANOVA was used; ns, not significant; *P<0.05; **P<0.01; ***P<0.0001.

The effects of diabetes on retinal thickness were measured using spectral domain optical coherence tomography (SD-OCT), as previously described (Pavlou et al., 2019; Sergeys et al., 2019). No difference was observed in the total retinal thickness between non-diabetic WT and *Il33^−/−^* mice at 6 months (Fig. S5A) and 9 months of age (Fig. 5A,B). However, non-diabetic *Il33^−/−^* mice at 9 months of age had thinner photoreceptor layers compared with those in age-matched WT counterparts (Fig. 5A,C). Diabetes caused a significant reduction of overall retinal thickness and photoreceptor layer thickness after 3 months (Fig. S5A,B) and 6 months (Fig. 5A-C) in WT mice compared with age-matched non-diabetic counterparts (Sergeys et al., 2019; Pavlou et al., 2019). The deletion of IL-33 further exacerbated the diabetes-induced retinal and photoreceptor layer thinning at 3 months (Fig. 5A,B) and 6 months of diabetes (Fig. 5A-C), compared with that in WT counterparts.

Immunohistochemistry showed no alteration in the number of cone arrestin (encoded by *Arr3*)^+^ cells between age-matched non-diabetic WT and *Il33^−/−^* mice (Fig. 5D,E). Induction of diabetes significantly reduced the number of cone photoreceptor cells in WT and *Il33^−/−^* mice after 6 months of diabetes (Fig. 5D,E), although the reduction was more profound in *Il33^−/−^* diabetic mice (Fig. 5D,E). The number of BRN3A (encoded by *Pou4f1*)^+^ ganglion cells in non-diabetic *Il33^−/−^* mice was comparable with that in WT counterparts (Fig. 6A,B). However, there was a significant loss of BRN3A^+^ ganglion cells in the retinas of both WT and *Il33^−/−^* mice after 6 months of diabetes, compared with that in their non-diabetic counterparts (Fig. 6A,B). Interestingly, the number of BRN3A^+^ cells in the *Il33^−/−^* diabetic retina was significantly lower than that in the WT counterpart (Fig. 6A,B). Taken together, our results suggest that IL-33 deficiency results in reduction of neuronal cells, including cone photoreceptors and ganglion cells, in the diabetic retina.

### IL-33 deficiency does not affect diabetes-induced acellular capillary formation

Acellular capillaries and endothelial cell death are early signs of retinal vasculopathy during diabetes (Sohn et al., 2016; Sergeys et al., 2019). Acellular capillaries were analysed by immunolabelling for collagen IV (basement membrane) and isolectin B4 (endothelium), and the vessels that were positive for collagen IV but negative for isolectin B4 were considered as acellular capillaries (Fig. 6C). The number of collagen IV^+^ isolectin B4^−^ acellular capillaries was significantly increased in the retinas of WT and *Il33^−/−^* diabetic mice compared with that in non-diabetic counterparts, and the number of acellular capillaries did not differ between the two strains of diabetic mice (Fig. 6C,D).

### IL-33 deficiency alters the expression of neurotransmitter and neurotrophin-related genes in the retina during diabetes

IL-33 is expressed predominately in Müller cells, which play an important role in the maintenance and survival of neurons by uptaking excessive neurotransmitters (glutamate and GABA), as well as synthesising various neurotrophins and providing energy substrates (glutamine and lactate) to neurons (Bringmann et al., 2009; Reichenbach and Bringmann, 2013; Simó et al., 2018). Recent studies have shown that an inflammatory milieu can trigger the production of neurotrophins in Müller cells, which in turn exert neuroprotective and anti-inflammatory effects to prevent neuronal cell death in the retina (Boss et al., 2017). Therefore, we investigated the impact of IL-33 deletion on Müller cell function by investigating the expression of neurotransmitter related genes and neurotrophins.

**Fig. 7.**
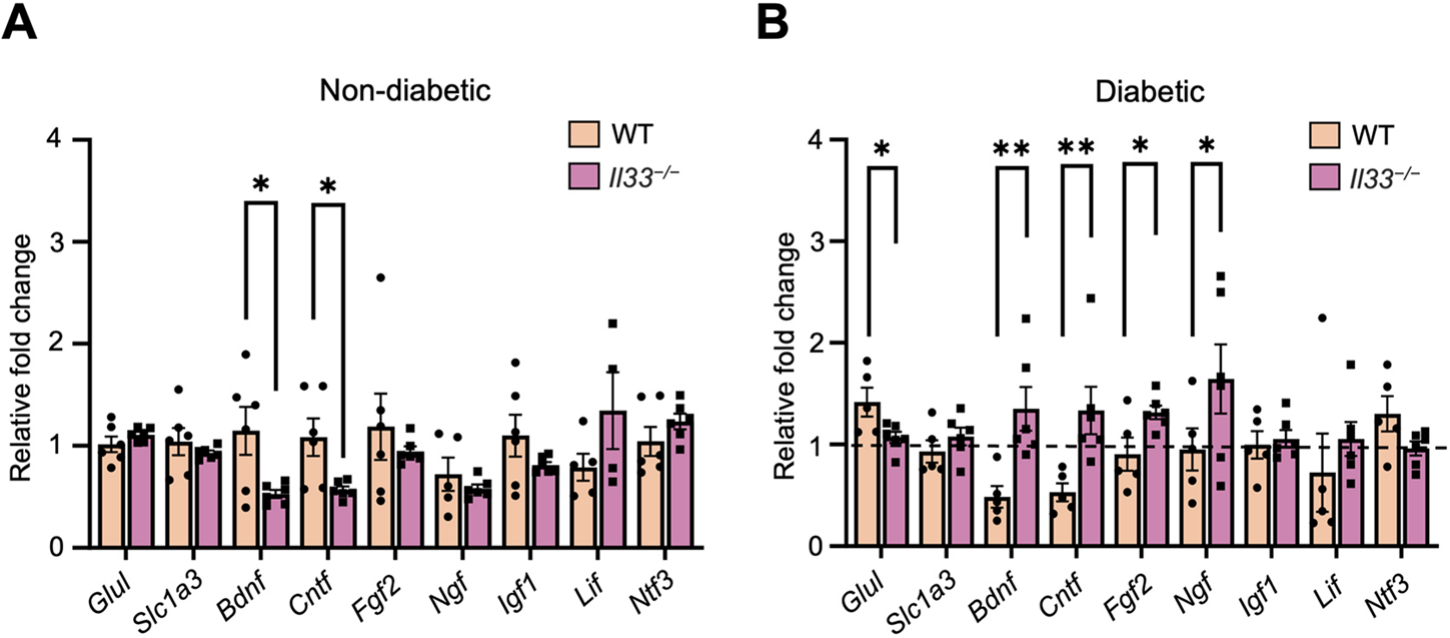
**Loss of IL-33 results in reduced expression of *Glul* and increased expression of neurotrophins in the retina after 6 months of diabetes.** (A) Expression levels of neurotransmitter-related genes and neurotrophins in WT and *Il33^−/−^* non-diabetic retinas, with significantly higher levels of *Bdnf* and *Cntf* in WT retinas. (B) Expression levels of neurotransmitter-related genes and neurotrophins in WT and *Il33^−/−^* diabetic retinas, compared with non-diabetic retinas from the same strain. Fold change values above the dotted line suggest increased response with diabetes and fold change values below the dotted line suggest decreased response with diabetes. WT diabetic retina expressed significantly higher levels of *Glul*, whereas *Il33^−/−^* diabetic retina expressed significantly higher levels of *Bdnf*, *Cntf*, *Fgf2* and *Ngf*. mRNA expression was assessed by RT-qPCR relative to *Rn18s* expression. *n*=5-6 retinas per experimental condition. Data show the mean±s.e.m. Unpaired two-tailed Student's *t*-test was used; *P<0.05; **P<0.01.

From the scRNA-seq analysis, we found increased expression of neurotrophins (*Bdnf*, *Lif* and *Igf1*) and activation markers (*Gfap* and *Vim*) in Müller cells of the diabetic retina compared with that in the non-diabetic retina (Fig. S2A,B). To investigate further, we compared the expression of glutamate metabolism-related genes and neurotrophins, such as *Glul*, *Slc1a3*, *Bdnf*, *Cntf*, *Fgf2*, *Ngf*, *Igf1*, *Lif* and *Ntf3*, in the non-diabetic and diabetic retinas of WT and *Il33^−/−^* mice (Fig. 7A,B). *Il33^−/−^* non-diabetic retinas had significantly lower levels of *Bdnf* and *Cntf* than the levels in the WT counterparts (Fig. 7A). However, after 6 months of diabetes, *Il33^−/−^* retinas expressed significantly lower levels of *Glul* and higher levels of *Bdnf*, *Cntf*, *Fgf2* and *Ngf* compared with the levels in WT retinas. The expression levels of *Slc1a3*, *Igf1*, *Lif* and *Ntf3* were comparable between WT and *Il33^−/−^* diabetic retinas (Fig. 7B).

### Effect of IL-33 deletion on the expression of neurotransmitter-related genes in primary Müller cells

**Fig. 8.**
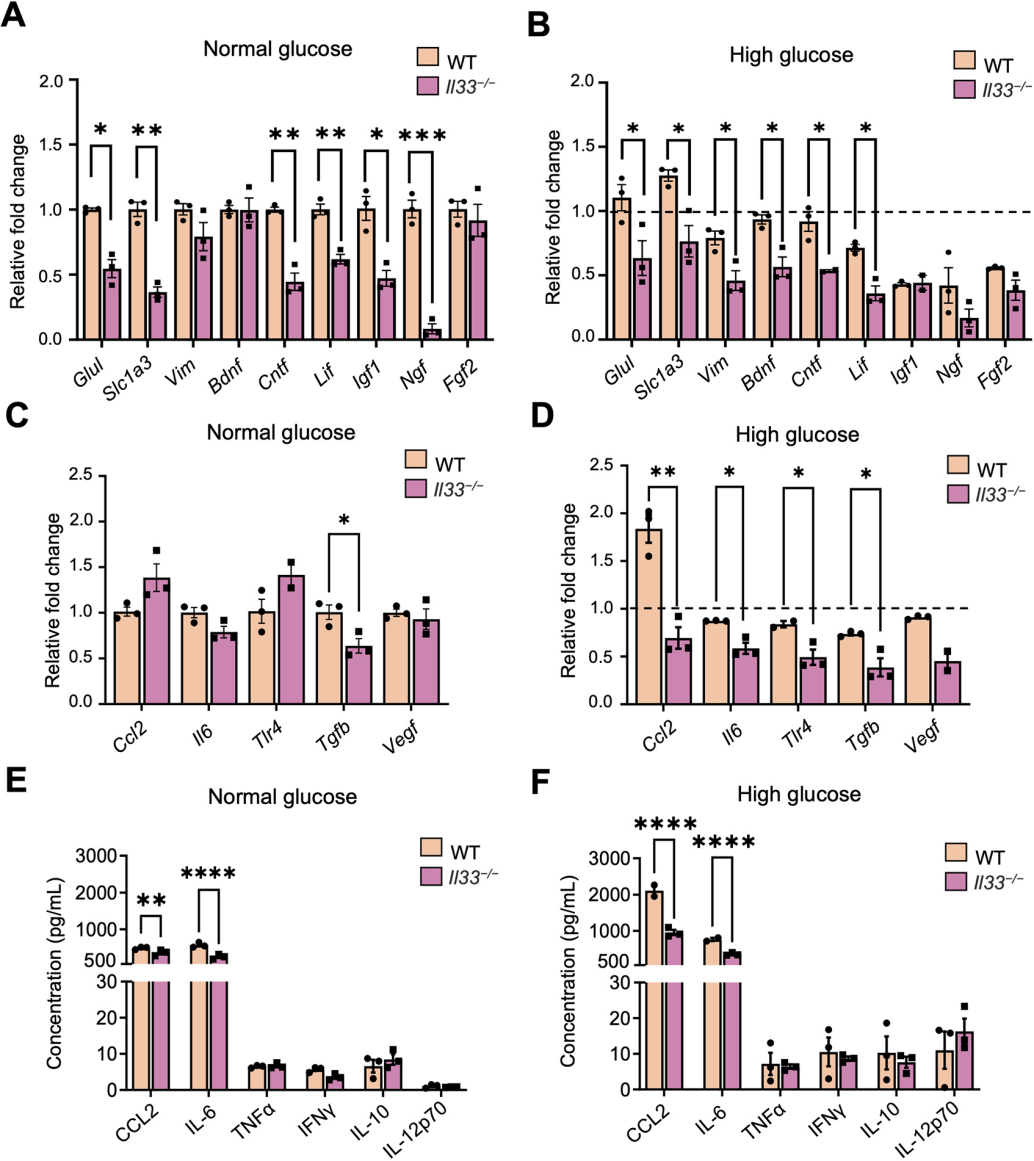
**PMCs from *Il33^−/−^* mice express lower levels of neurotransmitter-related genes, neurotrophins and inflammatory response genes after long-term high-glucose treatment.** (A,C) mRNA expression of neurotransmitter-related genes and neurotrophins (A) and inflammatory genes (C) in WT and *Il33^−/−^* PMCs treated with normal glucose. (B,D) mRNA expression of neurotransmitter-related genes and neurotrophins (B) and inflammatory genes (D) in WT and *Il33^−/−^* PMCs treated with high glucose, compared with that in PMCs treated with normal glucose from same strain. Fold change values above the dotted line suggest increased response with high glucose and fold change values below the dotted line suggest decreased response with high glucose. mRNA expression was assessed by RT-qPCR relative to *Rn18s* expression. (E,F) Cytokine secretion of CCL2, IL-6, TNFα, IFNγ, IL-10 and IL-12p70 in WT and *Il33^−/−^* PMCs treated with normal glucose (E) or high glucose (F). Data show the mean±s.e.m. *n*=3 per condition. Unpaired two-tailed Student's *t*-test (A-D,F) and two-way ANOVA (E) were used; *P<0.05; **P<0.01; ***P<0.001; ****P<0.0001.

Under normal glucose conditions, the expression levels of the neurotransmitter-related genes *Glul* and *Slc1a3*, and those of the neurotrophin genes *Cntf*, *Lif*, *Igf1* and *Ngf*, were significantly lower in primary Müller cells (PMCs) from *Il33^−/−^* mice than in cells from WT counterparts. The expression levels of *Vim*, *Bdnf* and *Fgf2* were comparable between WT and *Il33^−/−^* PMCs (Fig. 8A). The expression of other immune related genes including *Ccl2*, *Il6*, *Tlr4* and *Vegf*, were comparable between PMCs from WT and *Il33^−/−^* mice, although the expression of *Tgfb* was significantly lower in PMCs from *Il33^−/−^* mice (Fig. 8C). Interestingly, the production of CCL2 and IL-6 was significantly lower in PMCs from *Il33^−/−^* mice than in those from WT mice (Fig. 8E).

After long-term high glucose treatment, PMCs from WT mice expressed significantly higher levels of the neurotransmitter-related genes *Glul*, *Slc1a3* and *Vim*, and of the neurotrophin genes *Bdnf*, *Cntf* and *Lif*, compared with their levels in PMCs from *Il33^−/−^* counterparts (Fig. 8B). The expression levels of immune-related genes, including *Ccl2*, *Il6*, *Tlr4* and *Tgfb*, were significantly lower in PMCs from *Il33^−/−^* mice (Fig. 8D). The expression levels of *Vegf* (*Vegfa*) did not change between both mouse strains after high-glucose treatment (Fig. 8D). The production of CCL2 and IL-6 was significantly upregulated in PMCs from WT mice compared with that in PMCs from *Il33^−/−^* counterparts (Fig. 8F). Furthermore, the levels of TNFα, IFNγ (encoded by *Ifng*), IL-10 and IL-12 p70 (encoded by *Il12a* and *Il12b*) were comparable between WT and *Il33^−/−^* mice in both normal- and high-glucose conditions (Fig. 8E,F).

## DISCUSSION

The molecular mechanisms of DR are multifactorial and incompletely elucidated. It is established that progressive disruption occurs in the cell-to-cell communication within the retinal NVU during DR, which correlates with the severity of the disease. Neuroglial dysfunction and gliosis, deficits in electrophysiological function, and neurodegeneration leading to retinal thinning are amongst the earliest events occurring in clinical and experimental DR, which also represent several hallmarks of the disease (Simó et al., 2018; Antonetti et al., 2021). There is an urgent need to identify new targets to develop treatments for complexity of DR pathogenesis, as current therapies are not effective in all patients and do not address critical pathophysiology, such as early retinal neurodegeneration and inflammation (Stitt et al., 2016). With studies showing that sustained intraocular VEGF neutralisation results in neurodegeneration (Hombrebueno et al., 2015) but not vascular damage (Lechner et al., 2019) in the retina during diabetes, there is an unmet need to target pathways to prevent disease progression in the early stages of DR. Linked to these pathways, the importance of retinal inflammation in the pathogenesis of DR has become increasingly clear (Tang and Kern, 2011; Altmann and Schmidt, 2018; Semeraro et al., 2015; Okunuki et al., 2018). Amongst the altered factors that modulate retinal inflammation, IL-33 has been shown to play a key role in retinal neuron survival in a model of retinal detachment and regulate angiogenesis in laser-induced choroidal neovascularisation (Augustine et al., 2019; Clare et al., 2021). The precise role of IL-33 in Müller cells and regulation of inflammation in the retinal NVU during DR remains unknown.

In the current study, using human and murine scRNA-seq datasets, we confirmed that *Il33* is predominately expressed in retinal Müller cells. We found that the expression of *Il33* was increased under diabetic conditions, which provided a rationale to further investigate the role of IL-33 in DR. We also discovered that Müller cells and microglia are crucial in producing and secreting various inflammatory cytokines, growth factors and neurotrophins during DR to support retinal neurons. The deletion of IL-33 enhanced diabetes-induced retinal inflammation, evidenced by upregulated pro-inflammatory cytokine expression (*Ccl2*, *Il1b*, *Il6*, *Tnf*, *Tgfb* and *Inos*), sustained gliosis and microglia activation. Moreover, *Il33^−/−^* diabetic mice exhibit reduced electrophysiological function and developed severe retinal neuronal but not vascular degeneration. The expression of inflammatory mediators such as CCL2 and IL-6 was reduced in *Il33^−/−^* Müller cells under normal- and high-glucose conditions but increased in *Il33^−/−^* diabetic retina. These results suggest that IL-33 is critically involved in regulating diabetes-induced retinal inflammation and neurodegeneration.

Mechanistically, we found that IL-33 ablation reduced the expression of genes critical for glutamate metabolism, regulating extracellular levels of key neurotransmitters and secretion of neurotrophins in Müller cells. It has been shown that accumulation of glutamate is highly toxic to neurons and can stimulate the expression of neurotrophins (Barnett et al., 2001; Gionfriddo et al., 2009; Bringmann et al., 2013), as we saw in the diabetic retina of *Il33^−/−^* mice with significantly lower expression levels of *Glul* and higher expression levels of neurotrophins (*Bdnf*, *Cntf*, *Fgf2* and *Ngf*). In addition, high-glucose treatment of PMCs and the deletion of IL-33 led to reduced expression of neurotransmitter-related genes (*Glul*, *Slc1a3* and *Vim*) as well as neurotrophin genes (*Bdnf*, *Cntf* and *Lif*), which are crucial in glutamate clearance and neurotransmitter recycling. These results convey that IL-33 may play an unexplored role in Müller cell functions such as glutamate metabolism, uptake of neurotransmitters and production of neurotrophins (Gionfriddo et al., 2009). Thus, deletion of IL-33 in the retina during diabetes may disrupt glutamate clearance, neurotransmitter recycling and the secretion of neurotrophins (Barnett et al., 2001; Gionfriddo et al., 2009; Bringmann et al., 2013), and this leads to lower ERG a- and b-wave amplitudes, retinal neuronal thickness, and numbers of photoreceptors and ganglion cells during diabetes. We previously demonstrated that the reduction in ERG a- and b-wave amplitudes is a failure in the synaptic signals and dysfunction of neuronal activity in diabetic mice (Hombrebueno et al., 2014). Our results suggest that diabetes-mediated retinal neuronal damage is accelerated in *Il33^−/−^* mice.

Neurons are the primary cells that perform the core function of acquiring vision in the retina. When neurons are damaged, the supportive cells, i.e. vascular and glial cells, will react to remove insults and initiate repair to maintain retinal homeostasis (Xu and Chen, 2022). We detected significantly enhanced retinal microglial and Müller glial activation in *Il33^−/−^* diabetic mice. The increased glial activation could be the consequence of exaggerated diabetes-induced neuronal damage, in addition to IL-33 deficiency-mediated dysregulated retinal immune regulation. The chromatin binding of IL-33 is a key post-translational mechanism that regulates IL-33 release and bioactivity, with relatively high nuclear retention of IL-33 even within necrotic cells, and promotes a relatively slow release of IL-33 over time (Travers et al., 2018). We previously showed that, in a mouse model of retinal detachment, deletion of endogenous IL-33 accelerated the retinal degeneration accompanied by severe retinal inflammation. Macrophages from *Il33^−/−^* mice expressed significantly higher levels of *Inos*, *Tnf*, *Il1b* and *Ccl2*, with persistent inflammation in the retina possibly being related to the delayed clearance of inflammatory M1 macrophages or the lack of recruitment of or inability to induce anti-inflammatory M2 macrophages (Augustine et al., 2019). Interestingly, we detected lower levels of CCL2 and IL-6 expression/production in primary Müller cells from *Il33^−/−^* mice under hypoxic conditions (Augustine et al., 2019). In line with our previous study, in the current study, we detected lower levels of CCL2, IL-6 and TGFβ in primary Müller cells from *Il33^−/−^* mice under normal- and high-glucose conditions. Our results suggest that IL-33 is critical for the immunomodulatory function of Müller cells. The crosstalk between Müller cells and microglia in retinal pathophysiology is well appreciated (Wang et al., 2011; Inada et al., 2021). Collectively through distinct mechanisms, IL-33 potentially functions as a nuclear effector to maintain and regulate inflammatory cytokine expression and clearance of glutamate in the retina during diabetes.

There is increasing evidence showing the homeostatic nature of IL-33 in the eye and brain, through modulating the immune response and mediating metabolism and autophagy, which provides an exciting avenue of research to target the complex disease pathways involved in DR (Theodoropoulou et al., 2017; Barbour et al., 2014; Augustine et al., 2019; Clare et al., 2021). IL-33 levels were significantly upregulated in the vitreous of patients with AMD compared with those in individuals without AMD (Xi et al., 2016). However, the expression of *Il33* remained unchanged in the retina and significantly reduced in the RPE/choroid/sclera of patients with AMD (Kim et al., 2018; Clare et al., 2021). It has been shown that endogenous IL-33 is a critical regulator of metabolism in RPE cells and is essential for utilisation of pyruvate in the tricarboxylic acid cycle and mitochondrial metabolism, through inducing the abundance of mitochondrial pyruvate carrier 1 (MPC1). In addition, RPE cells become vulnerable to oxidative stress upon deletion of IL-33, as they undergo more aerobic glycolysis than mitochondrial respiration, subsequently leading to the development of conditions permissive for AMD pathogenesis (Scott et al., 2021). Deficiency of IL-33 also leads to a reduction in a central autophagy protein, LC3II, in the brain and ovarian follicle tissues, demonstrating the important role for IL-33 in maintaining autophagy (Wu et al., 2015). Administration of IL-33 has been shown to modulate the innate immune response by polarising microglia/macrophages towards an anti-inflammatory phenotype and reducing the expression of proinflammatory genes (*Il1b*, *Il6* and *Nlrp3*) in the cortices of APP/PS1 mice, an experimental model of Alzheimer's disease (Fu et al., 2016). During central nervous system injury, mice lacking IL-33 have been shown to have impaired recovery owing to reduced myeloid cell infiltrates and decreased induction of M2 genes at the injury site (Gadani et al., 2015). Together, the IL-33 pathway and potential of IL-33 gene therapy warrants further investigation as a possible therapeutic avenue in modulating immune dysregulation for the treatment of DR.

Overall, our study uncovered a significant role of IL-33 in regulating glia-neuron crosstalk in the retina. The upregulation of IL-33 in Müller cells during diabetes is a countermeasure seeking to support damaged neurons and restore impaired retinal NVU by regulating glutamate metabolism, neurotransmitter recycling and the secretion of neurotrophins. IL-33 plays an important regulatory role in glial-mediated retinal diseases, and bolstering the endogenous protective responses of IL-33 in Müller cells may be a novel approach to manage early-stage DR.

## MATERIALS AND METHODS

### ScRNA-seq analysis

ScRNA-seq datasets of human and murine retinas were used from the Gene Expression Omnibus database with the accession numbers GSE196235 and GSE178121, respectively (Sun et al., 2021; Wang et al., 2022). Normalisation, dimensionality reduction and clustering of single cells were performed in R version 4.1.0 using Seurat version 4. Cells expressing fewer than 250 genes and more than 20% of mitochondrial genes were filtered out. Cells were clustered by their gene expression profiles using the graph-based clustering algorithm. This approach consists of building a sparse k-nearest-neighbour (KNN) graph with edges drawn between cells of similar feature expression patterns, followed by Louvain modularity optimisation.

### Animals and induction of diabetes

*Il33^−/−^* mice on the C57BL/6N background (Oboki et al., 2010) were obtained from RIKEN Center for Life Science Technologies, Japan (accession number CDB0631K) and were cross-bred with C57BL/6J (WT) mice to eliminate the *Rd8* mutation as described previously (Augustine et al., 2019). *Il33^−/−^* mice were confirmed to be negative for *Rd8* mutation during genotyping of new litters. Both WT and *Il33^−/−^* mice were housed in a 12-h/12-h light/dark cycle condition with unlimited access to food and water in the Biological Service Unit at Queen's University Belfast. All *in vivo* experiments were approved by the Animal Welfare Ethical Review Body (AWERB) of Queen’s University Belfast. The protocol complied with the UK Home Office Animals (Scientific Procedures) Act 1986, with compliance with the Association for Research in Vision and Ophthalmology (ARVO) statement for the use of animals in ophthalmology and vision research.

Diabetes was induced in 3-month-old male WT and *Il33^−/−^* mice (∼25 g) by five daily intraperitoneal injections of 50 mg/kg STZ (Sigma-Aldrich, MI, USA) in freshly prepared 0.1 M citrate buffer (pH 4.5), as previously described by our group (Pavlou et al., 2019). Age-matched non-diabetic mice were injected with citrate buffer. One week after injections, hyperglycaemia was measured using the FreeStyle Lite Blood Glucose monitoring kit (Abbott Laboratories, IL, USA), and mice with levels >15 mM were designated as diabetic. The mouse experimental groups were maintained for up to 6 months and were euthanised by CO_2_ asphyxiation, after which blood was collected to assess glycated haemoglobin (HbA1c) levels using the A1cNow^+^ kit (PTS Diagnostics, IN, USA), according to the manufacturer's instructions.

### Retinal assessment

Ganzfeld electroretinography responses were evaluated on all mice at 3 and 6 months post diabetes induction using the Espion visual electrophysiology system (Diagnosys Technologies, MA, USA) following the manufacturer's guidelines and as previously described (Augustine et al., 2019; Pavlou et al., 2019). Briefly, eight light intensities ranging from 0.008 to 25 cd.s/m^2^ were applied and the averages of four responses for each light intensity were recorded for a- and b-waves of each mouse. Oscillatory potentials (OPs) were recorded using 25 cd.s/m^2^ light intensity. The a- and b-wave amplitudes, implicit times and OPs were calculated using Espion analysis software based on the manufacturer's calibrations (Diagnosys Technologies).

SD-OCT images (30° field of view) were collected from all the experimental groups at 3 and 6 months of diabetes using the Spectralis Heidelberg OCT system (Heidelberg Engineering, Germany), according to the manufacturer's instructions and as previously described (Harkin et al., 2022). Total neural retinal and photoreceptor layer thickness were measured at 1500 μm eccentricities from the optic disk in the four quadrants of retina (nasal, temporal, superior and inferior) using the Spectralis Heidelberg OCT analysis software (Heidelberg Engineering).

Fundus images of retina were acquired from all the experimental groups at 6 months of diabetes using a Micron IV mouse fundus camera (Phoenix Research Laboratories, OR, USA) according to the manufacturer’s instructions and as previously described (Augustine et al., 2019).

### Immunohistochemistry and analysis

The eyes from all mouse experimental groups were collected at 6 months post diabetes induction and fixed in 2% paraformaldehyde (Sigma-Aldrich) for 2 h at room temperature for immunostaining on retinal cryosections and flatmounts. Immunostaining was performed on 14 µm-thick cryosections (Leica CM1900 cryostat, Leica Microsystems, UK) following previously described protocols (Augustine et al., 2019). Primary and secondary antibodies listed in Table S1 were used, and a negative control without the primary antibody was conducted for each staining. Images were acquired using a Nikon Eclipse 80i fluorescence microscope, a Nikon Eclipse TE200-U C1 laser scanning confocal microscope (Nikon, Tokyo, Japan) and a Leica SP8 laser scanning confocal microscope (Leica Microsystems, Wetzlar, Germany) with the same settings for each primary antibody. Images were processed using Fiji software (provided in the public domain). The numbers of microglia, cone-arrestin^+^ photoreceptor cells and BRN3A^+^ ganglion cells were counted using the multi-point tool in Fiji software. GFAP quantification was achieved by counting GFAP^+^ fibres within the INL of the retina (Augustine et al., 2019). The values were averaged from two sections (six images) per retina and the data were normalised to 100 µm of retinal length.

Immunostaining was performed on retinal flatmounts following previously described protocols (Pavlou et al., 2019). Primary and secondary antibodies listed in Table S1 were used, and a negative control without the primary antibody was conducted for each staining. Images were acquired using the Nikon Eclipse TE200-U C1 confocal microscope and the number of acellular capillaries were counted in the superficial, intermediate and deep plexus layers of the retina. The values were averaged from eight images per retina (two images per retinal quadrant) and the data were normalised to per unit area of retinal tissue in mm^2^.

### Cell culture experiments and high-glucose treatment

PMCs were cultured from the retinas of WT and *Il33^−/−^* pups at postnatal day 7, as previously described (Augustine et al., 2019, 2018). Briefly, PMCs were cultured in complete DMEM (low-glucose; 22320022, Thermo Fisher Scientific, MA, USA) supplemented with 10% heat-inactivated fetal calf serum (FCS; 10270106, Thermo Fisher Scientific) and 1% Penicillin-Streptomycin (15140122, Thermo Fisher Scientific). They were routinely tested for mycoplasma contamination by PCR. WT and *Il33^−/−^* PMCs were treated with 5 mM normal glucose (standard DMEM supplemented with 25 mM D-mannitol for osmotic control, Sigma-Aldrich) or 30 mM high glucose (standard DMEM supplemented with 25 mM D-glucose, Sigma-Aldrich) for 2 weeks, following previously described protocols (Llorián-Salvador et al., 2020; Albert-Garay et al., 2022). The culture medium was changed during passaging of cells. PMCs were then lysed for RT-qPCR and supernatants were collected for cytokine bead array.

### RNA isolation and RT-qPCR

Total RNA from mouse neuroretinas and cell lysates was extracted using the RNeasy Mini Kit (QIAGEN, Hilden, Germany) and the same amount of RNA was transcribed into cDNA using the SuperScript II Reverse Transcriptase Kit (Thermo Fisher Scientific) following the manufacturer's instructions. Transcript levels of genes were analysed by RT-qPCR performed using a Roche LightCycler 480 (Roche, Basel, Switzerland) with either TaqMan assays or SYBR Green detection as previously described (Harkin et al., 2022; Augustine et al., 2018). Relative gene expression was calculated using the comparative Ct method (2^−ΔΔCt^) with data normalised to *Actb* or *Rn18s*. Roche-validated mouse TaqMan assays were purchased for *Ccl2* (310467), *Il1b* (310471), *Il18* (301115), *Il33* (316824), *Il6* (300699), *Tgfb* (317139), *Tnf* (317484), *Vegf* (314944), *Actb* (307903) and *Rn18s* (307906). SYBR Green gene-specific primers were designed as previously described (Augustine et al., 2018) and the primer sequences are listed in Table S2.

### Cytokine quantification

Supernatants from cultures of PMCs were measured for CCL2, IL-6, TNFα, IFNγ, IL-10 and IL-12p70 levels using Cytokine Bead Array Flex sets (BD Biosciences, NJ, USA) following the manufacturer's guidelines and as previously described (Augustine et al., 2019). The BD FACS Canto II flow cytometer was used to assess the samples and data were analysed using FCAP ArrayTM Software (BD Biosciences).

### Statistical analysis

Double-anonymised quantification was conducted by two researchers for the analysis and the results of datasets were plotted either in line graphs or bar plots to show individual data points and mean±s.e.m. Statistical analysis was performed using Prism 9 (GraphPad Software, CA, USA). Normality and equal variance were assessed prior to use of unpaired two-tailed Student's *t*-test (with or without Welch's correction) or two-way ANOVA with Sidak's post hoc correction for multiple comparisons. *P*<0.05 was considered statistically significant.

## Supplementary Material

10.1242/dmm.050174_sup1 Supplementary information
