# Supplementary material for: IL-33 regulates Müller cell-mediated retinal inflammation and neurodegeneration in diabetic retinopathy
Source: Dis Model Mech. 2023 Sep 6;16(9):dmm050174. doi: 10.1242/dmm.050174 (PMC10499035; doi:10.1242/dmm.050174)
Supplement: Supplementary information [file dmm-16-050174-s1.pdf]

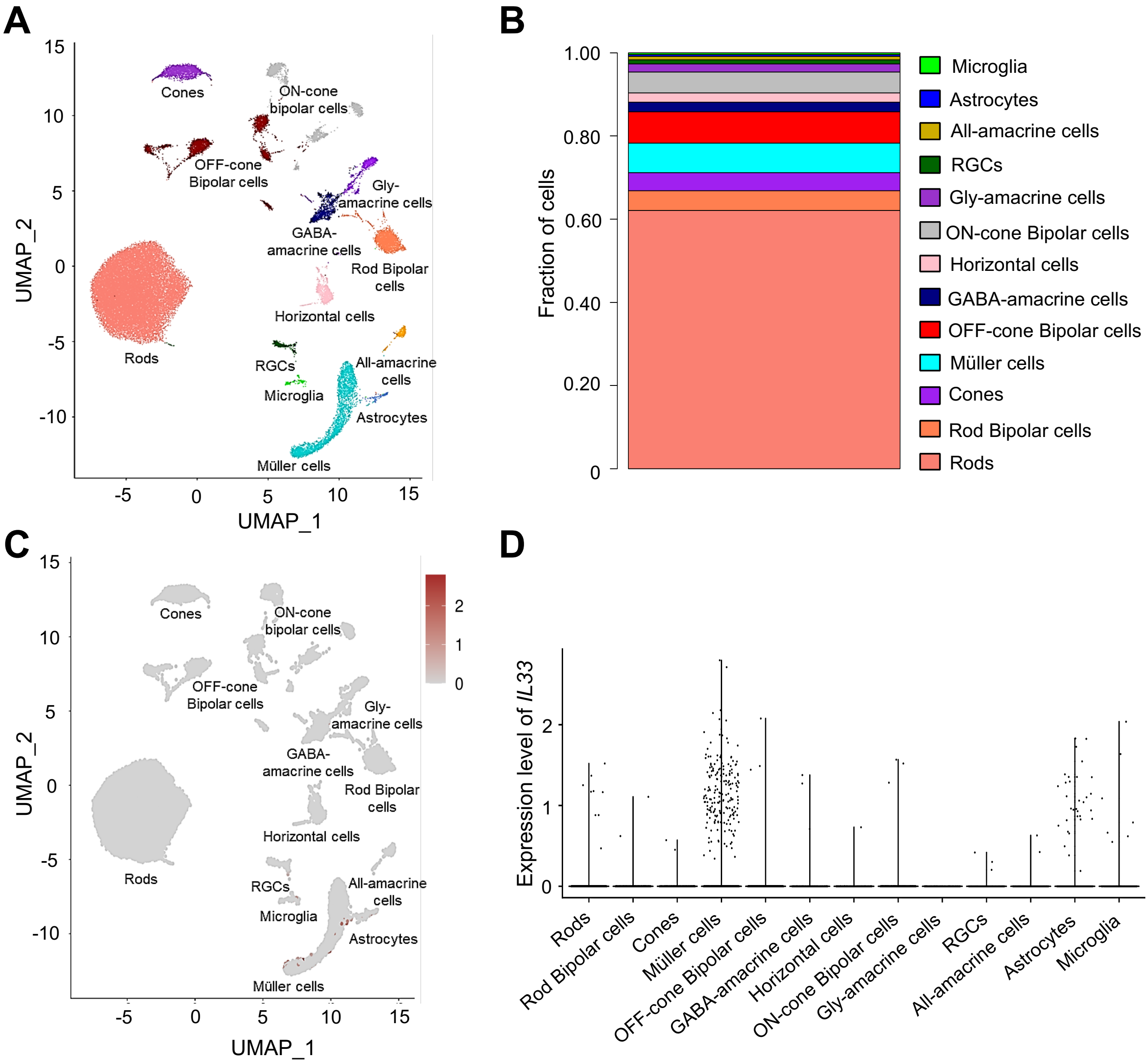

Fig. S1. Expression of *IL33* in non-diabetic human retina. (A) ScRNA-seq showing the UMAP plot of 22 clusters were resolved and assigned to 13 cell types. (B) Fraction of different cell types in the non-diabetic human retina as determined by scRNA-seq, calculated from absolute counts out of 51,645. (C) The global expression changes of *IL33* is exhibited in UMAP plot. (D) Violin plots display the dominant expression of *IL33* in the Müller cell cluster of retinal cells.

Disease Models & Mechanisms • Supplementary information

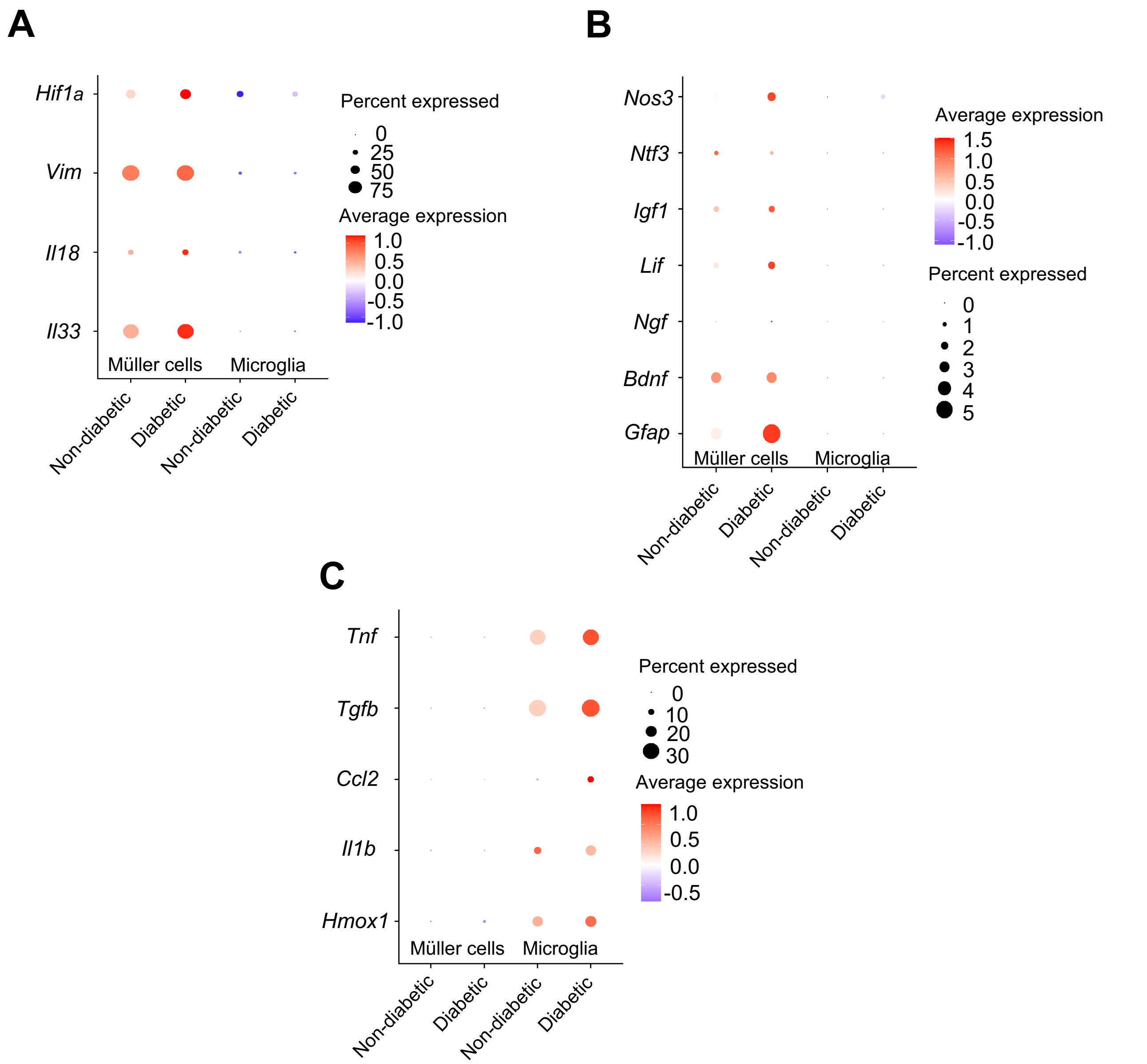

Fig. S2. The pathological changes of Müller cells and microglia in the WT retina after 6 months of diabetes. (A) Bubble plots exhibit the dominant expression of *Il33, Il18, Vim,* and *Hif1a* in Müller cells during diabetes. (B) Bubble plots display the dominant expression of *Gfap, Bdnf, Ngf, Lif, Igf1, Ntf3*, and *Nos3* in Müller cells during diabetes. (C) Bubble plots demonstrate the dominant expression of *Hmox1, Il1b, Ccl2, Tgfb*, and *Tnf* in microglia during diabetes.

Disease Models & Mechanisms • Supplementary information

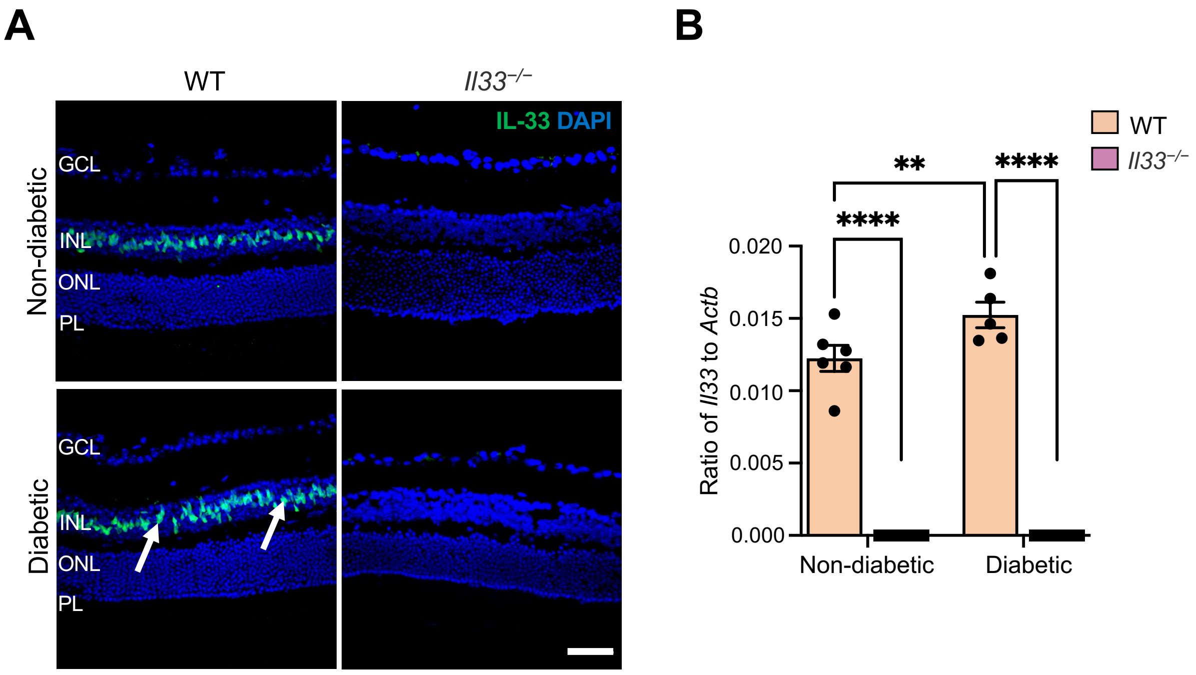

Fig. S3. Expression of IL-33 in the retinas of WT and *Il33*$^{-/-}$ mice after 6 months of diabetes. (A) Representative images of retinal cryosections from WT and *Il33*$^{-/-}$ non-diabetic and diabetic mice immunostained for IL-33 (green, arrows) and counterstained with DAPI (blue). GCL, ganglion cell layer; INL, inner nuclear layer; ONL, outer nuclear layer; PL, photoreceptor layer. Scale bar: 50 μm. (B) Bar graph showing *Il33* mRNA expression in non-diabetic and diabetic retinas from WT and *Il33*$^{-/-}$ mice as a ratio to *Actb* expression, assessed by RT-qPCR. *Il33* expression of WT non-diabetic and diabetic retinas in this data are also plotted in Fig. 1G, as relative to *Actb* expression. *n*=5-6 mice per experimental group. Data show the mean±s.e.m. Two-way ANOVA was used; ns, not significant; ***P*<0.01; *****P*<0.0001.

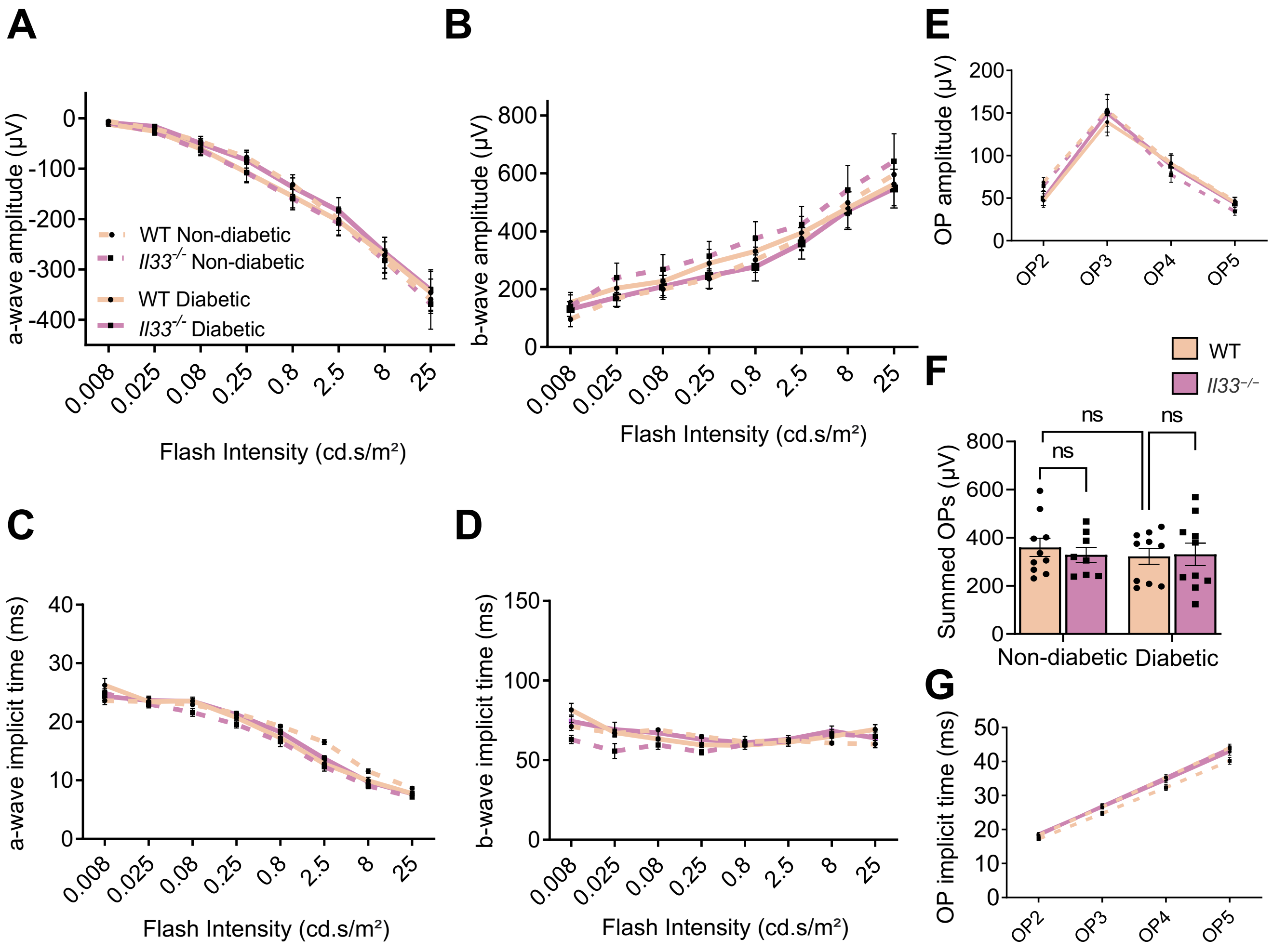

Fig. S4. IL-33 deficiency exhibits no alteration of ERG a- and b-wave response in the retina after 3 months of diabetes. (A-D) Line graphs of average scotopic ERG a-wave (A), average scotopic ERG b-wave (B), average scotopic ERG a-wave implicit time (C) and average scotopic ERG b-wave implicit time (D) in the eyes of WT and *Il33*$^{-/-}$ non-diabetic and diabetic mice, quantified from 0.008 cd.s/m^2 to 25 cd.s/m^2. (E,G) Line graphs of average oscillatory potential (OP) amplitudes (E) and average OP implicit times (G) in the eyes of WT and *Il33*$^{-/-}$ non-diabetic and diabetic mice, quantified at 25 cd.s/m^2. (F) Bar graph of summed OP amplitudes in the eyes of WT and *Il33*$^{-/-}$ non-diabetic and diabetic mice, quantified at 25 cd.s/m^2. *n*=4-5 mice per experimental group. Data show the mean±s.e.m. Two-way ANOVA was used; ns, not significant.

Disease Models & Mechanisms • Supplementary information

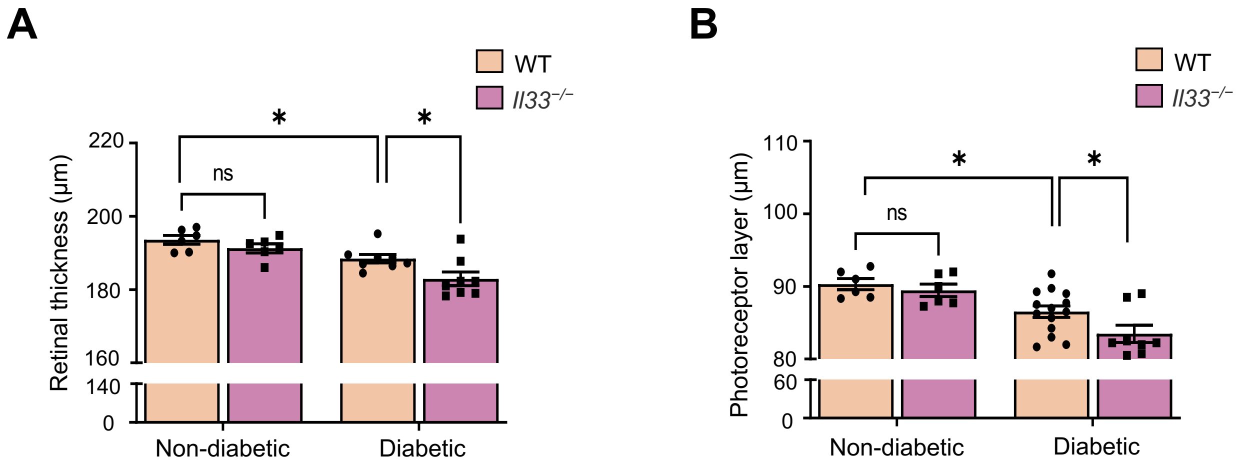

Fig. S5. Deletion of IL-33 results in reduced retinal and photoreceptor layer thickness in the retina after 3 months of diabetes. (A,B) Bar graph showing the average total retinal thickness (A) and average photoreceptor layer thickness (B) in the retinas of WT and *Il33*$^{-/-}$ non-diabetic and diabetic mice. *n*=4-6 mice per experimental group. Data show the mean±s.e.m. Two-way ANOVA was used; ns, not significant; **P*< 0.05.

Table S1. Antibodies used for immunohistochemistry. Primary and secondary antibodies listed by name, dilution, manufacturer, and catalogue number.

Antibody	Dilution	Manufacturer	Catalogue No.
BRN3A	1:200	Santa Cruz	SC-31984
Collagen IV	1:75	Bio-Rad	2150-1470
Cone arrestin	1:1000	Sigma-Aldrich	AB15282
F4/80	1:100	Abcam	AB6640
GFAP	1:200	DAKO	Z0334
IBA1	1:100	Invitrogen	PA5-18039
IL-33	1:50	R&D systems	AF3626
Isolectin B4	1:50	Vector labs	B-1205
P2RY12	1:100	AnaSpec	ANA55043A
Donkey anti-goat IgG Alexa Fluor 488	1:300	Jackson ImmunoResearch	705-545-147
Donkey anti-goat IgG Alexa Flour 568	1:300	Invitrogen	A-11057
Donkey anti-rabbit IgG Alexa Fluor 488	1:300	Jackson ImmunoResearch	711-545-152
Donkey anti-rabbit IgG Alexa Fluor 594	1:300	Jackson ImmunoResearch	711-585-152
Donkey anti-rat IgG Alexa Fluor 594	1:300	Invitrogen	A-11007
Donkey anti-rat IgG Alexa Flour 647	1:300	Jackson ImmunoResearch	712-605-150
Streptavidin Alexa Fluor 488	1:300	Invitrogen	S32354

Table S2. Primers used for RT-qPCRs. Forward and reverse primer sequences of the targeted mouse genes were purchased from Integrated DNA Technologies.

Gene	Forward primer	Reverse primer
Bdnf	GGGTCACAGCGGCAGATAAA	GCCTTTGGATACCGGGACTT
Cntf	AGCCTTGACTCAGTGGATGG	TGGAGGTTCTCTTGGAGTCG
Fgf2	CAACCGGTACCTTGCTATGA	TCCGTGACCGGTAAGTATTG
Glul	TGCCTGCCCAGTGGGAATT	TATTGGAAGGGTTCGTCGCC
Igf1	AGACAGCATTGTGGATGAG	TGAGTCTTGGGCATGTCAGT
Inos	GGCAAACCCAAGGTCTACGTT	TCGCTCAAGTTCAGCTTGGT
Lif	AATGCCACCTGTGCCATACG	CAACTTGGTCTTCTCTGTCCCG
Ngf	GGCGTACAGGCAGAACCGTA	CAGCCTCTTCTTGTAGCCTTC
Ntf3	AGGTGATGTCCATCTTGTTTT	GCCTCTCCCTGCTCTGGTTC
Slc1a3	CTCTGGGCATCCTCTTCTG	CAAATCTGGTGATGCGTTTG
Tlr4	TCCTGGCTAGGACTCTGATCAT	TCCAGCCACTGAAGTTCTGA
Vim	GTACAAGTCCAAGTTTGCTG	ATCGTGATGCTGAGAAGTCT
Actb	CCTTCCTTCTTGGGTATG	TGTAAAACGCAGCTCAGTAA
Rn18s	AGGGGAGAGCGGGTAAGAGA	GGACAGGACTAGGCGGAACA

Disease Models & Mechanisms • Supplementary information
